# Role of Lipoprotein(a) Reduction in Cardiovascular Disease

**DOI:** 10.3390/jcm13216311

**Published:** 2024-10-22

**Authors:** Uma Schuth, Kieran Gill, Pyotr Telyuk, Bilal-Reshad Bawamia, David Austin, Azfar Zaman

**Affiliations:** 1 Faculty of Medicine, St Andrews University, St Andrews KY16 9ST, UK; uma_schuth@btconnect.com; 2 Translational and Clinical Research Institute, Faculty of Medical Sciences, Newcastle University, Newcastle upon Tyne NE2 4AX, UK; k.gill3@newcastle.ac.uk; 3 Academic Cardiovascular Unit, The James Cook University Hospital, South Tees Hospitals NHS Foundation Trust, Middlesbrough TS4 3BW, UK; p.telyuk@nhs.net (P.T.); david.austin@nhs.net (D.A.); 4 Cardiology, Freeman Hospital, Newcastle upon Tyne NE7 7DN, UK; bilal-reshad.bawamia@nhs.net; 5 Population Health Sciences Institute, Newcastle University, Newcastle-upon-Tyne NE2 4AX, UK; 6 Cardiology, Freeman Hospital and Vascular Biology and Medicine, Newcastle University, Newcastle upon Tyne NE2 4AX, UK

**Keywords:** lipoprotein(a), cardiovascular disease, atherosclerosis

## Abstract

Recent studies have shown that lipoprotein(a) (Lp(a)) is an important risk factor for a plethora of different cardiovascular diseases. It has been proven that Lp(a) levels are genetically determined and correlate with risk of cardiovascular disease, independent of lifestyle factors. As of yet, treatment options to reduce Lp(a) levels are limited, but new research into Lp(a) reduction yields promising results. This review delves into Lp(a)’s biochemistry and mechanism of effect, the association between Lp(a) and cardiovascular diseases, and possible therapies to minimise cardiovascular disease.

## 1. Introduction

Lipoprotein(a) has emerged as an independent and causal risk factor for atherosclerotic cardiovascular disease (CVD) and calcific aortic stenosis through mechanisms associated with atherogenesis, inflammation, and thrombosis. Multiple large-scale, prospective epidemiological studies demonstrate a robust association between elevated Lp(a) levels and an increased incidence of ischaemic heart disease (IHD), myocardial infarction (MI), stroke, and peripheral vascular disease. Further population-based data identified an association of increased Lp(a) with the incidence and rate of progression of calcific aortic stenosis. The value of these associations lies in their independence from traditional cardiovascular risk factors, including diabetes, hypertension, and smoking. Lp(a) levels are largely determined by genetic factors, with minimal influence from dietary or other factors. The therapeutic effects of established cardiovascular treatment strategies on Lp(a) levels are uncertain and do not result in optimal Lp(a) reduction. This review highlights the important role of Lp(a) in CVD.

## 2. Lipoprotein(a) Biochemistry

**Figure 1:**
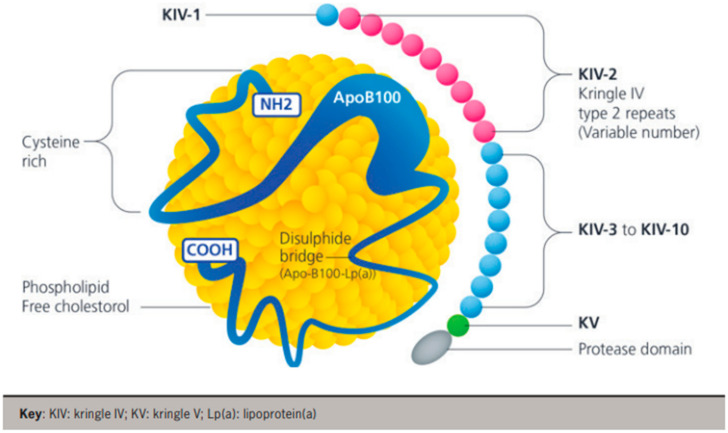
Lipoprotein(a) (Lp[a]) particle containing apolipoprotein B100 and apolipoprotein(a).

The structure of lipoprotein(a) [Lp(a)] (Figure 1) consists of two parts: (i) a large, hydrophilic apolipoprotein(a) [apo(a)] glycoprotein and (ii) an LDL-like particle containing an ApoB100 glycoprotein on its surface [1]. The apo(a) glycoprotein is bound to the ApoB100 glycoprotein by a single disulfide bond, and the presence of this apo(a) glycoprotein differentiates Lp(a) from LDL [1]. Apo(a) is genetically determined (encoded by the Lp(a) gene on chromosome 6) in an autosomal co-dominant pattern of inheritance [2].

Within the apo(a) molecule are repeated domains called kringles. Polymorphisms within the hypervariable apo(a) gene arise due to varying numbers of kringle IV type 2 (IV_2_) repeats [3,4], resulting in over forty apo(a) isoforms. This finding is clinically relevant, as smaller apo(a) isoforms (with fewer kringle IV_2_ repeats) are associated with a higher plasma concentration of Lp(a) and an increased incidence of coronary heart disease, ischaemic stroke, and calcific aortic stenosis [5,6]. Lp(a) plasma concentration exhibits high heritability and factors such as age, sex, diet, and exercise seemingly exert little influence on Lp(a) concentrations [6]. The variation in kringle IV_2_ repeats makes accurate measurement of plasma Lp(a) concentrations difficult [4].

## 3. Lipoprotein(a) Biology and Genetic Variability

Amino acid sequencing of apo(a) shows remarkable structural similarity to plasminogen [7], with the kringle IV_2_ repeats of apo(a) being highly homologous to the kringle IV domains of the plasminogen gene. Phylogenetic analysis showed that the LP(a) gene evolved from the plasminogen gene, with Lp(a) only found in humans and hedgehogs [8]. An evolutionary explanation as to why humans evolved to produce high concentrations of Lp(a) is unclear, but research suggests Lp(a) provided a survival advantage through enabling thrombosis, which is important in wound healing and increasing haemostasis during parturition as well as reducing major haemorrhage in the brain and airways [9]. In normal physiology, Lp(a) transports cholesterol esters and triglycerides in the bloodstream from the liver to peripheral tissues to help maintain lipid homeostasis in the endogenous lipoprotein pathway. Lp(a) is synthesised and metabolised by the liver, but whether Lp(a) is cleared by the LDL receptor is unknown [10].

There is extensive variability in Lp(a) levels between individuals and populations that cannot be fully explained by genetic factors alone. Randomised controlled clinical trials show that diets lower in saturated fats only modestly influence Lp(a) levels, and often in the opposing direction to LDL cholesterol. The effect of physical activity/exercise is inconsistent, ranging from no change to moderate change in Lp(a) levels. However, this variation is likely modulated by age and the type, intensity, and duration of exercise undertaken. Of interest, hormone replacement therapy (HRT) in postmenopausal women has been shown to lower Lp(a) levels, with oral being more effective than transdermal estradiol; the type of HRT, dose of oestrogen, and addition of progestogen do not modify the Lp(a)-lowering effect of HRT. Kidney diseases also result in modulation of Lp(a) levels, with increased levels seen in advancing disease stages, dialysis-type, and apolipoprotein(a) phenotypes. In contrast, Lp(a) levels are reduced in liver diseases in parallel with liver disease progression, although population studies have yielded conflicting results on the associations between Lp(a) levels and nonalcoholic fatty liver disease. Overall, current evidence supports the role of diet, hormones, and chronic liver and kidney diseases in modifying Lp(a) levels [11].

Lp(a) is regulated mainly genetically by the LPA gene, but involved genetic variants have not been fully elucidated. Improved understanding of the entanglements of genetic Lp(a) regulation may enhance genetic prediction of Lp(a) and CAD risk. Lp(a) concentrations are believed to be largely controlled by one single gene (LPA) that contains a complex interplay of several genetic elements with many effects. The effects of the apo(a) isoforms are, however, modified by many functional single-nucleotide polymorphisms (SNPs) distributed over the complete range of allele frequencies (<0.1% to >20%), with pronounced effects on Lp(a) concentrations. A complex interaction is present between the apo(a) isoforms and LPA SNPs. Differences in the Lp(a) trait between ancestries may be caused by differences in the frequency and the isoform association of multiple LPA SNPs, with a large impact on Lp(a) concentrations and Lp(a) distribution. However, environmental exposures and inflammatory burden have also been proposed as causal factors. A comprehensive catalogue of the functional genetic variation in LPA across ancestries remains elusive but is needed to define the true remainder, which may then be attributable to polygenic influences or environment [12].

## 4. Mechanism of Lipoprotein(a) Effect (Appendix A Figure A1)

Whilst plasminogen can be broken down by tissue plasminogen activator (tPa), urokinase, or streptokinase to form plasmin that degrades clots, apo(a) is resistant to proteolysis due to a single amino acid substitution in the apo(a) gene [1], resulting in an inactive protease domain. This gives apo(a) pro-thrombotic properties as it competes with plasminogen to bind to fibrin, but apo(a) does not mediate fibrinolysis. Smaller apo(a) isoforms have a higher affinity for fibrin, supporting their higher thrombogenic potential [10]. When this occurs at sites of plaque rupture, this may cause myocardial infarction (MI) and ischaemic stroke. Lp(a) also contains oxidised phospholipids (oxPLs) [6], which co-localise with apo(a) in the arterial tunica intima and aortic valve annulus. This promotes endothelial dysfunction (leading to the proliferation of vascular smooth muscle cells), increases macrophage apoptosis, causing Lp(a) to accumulate in the arterial wall as well as increasing calcification [13]. These mechanisms contribute to the pathogenesis of atherosclerosis and calcific aortic stenosis. This could also explain why Lp(a) is around six times more atherogenic than LDL on a per-particle basis [14].

## 5. Association Between Elevated Lp(a) and Cardiovascular Disease

There is emerging consensus from population studies about the effect of Lp(a) levels on cardiovascular disease risk—there being a consistent correlation between Lp(a) levels and risk of atherosclerotic cardiovascular disease (ASCVD) (Table 1) [15]. However, the cardiovascular diseases studied have shared commonalities in their pathogenesis. Lp(a) has been shown to have a baseline residual effect on the risk of atherosclerotic cardiovascular disease that currently cannot be fully compensated for purely through lifestyle changes [16]. A meta-analysis of twenty-seven international prospective studies identified “a clear association between Lp(a) and” [17] ASCVD, showing the baseline risk of ASCVD in the top third of Lp(a) concentrations to be 1.7 times higher than in the lowest third [17]. Further epidemiological data showed a relationship between higher Lp(a) levels and the incidence of MI. The study involved over 12,000 patients stratified by ethnicity and adjusted for age and sex. The association of elevated Lp(a) concentration with new MI was independent of cardiovascular risk factors, including diabetes, hypertension, and smoking. This observational study also demonstrated an inverse association between isoform size and Lp(a) concentration, indicating a lower risk of MI with higher isoform size [15]. Observational data also suggest increased prevalence of recurrent ischaemic events and rates of repeat revascularisation in patients with previous percutaneous coronary intervention (PCI) [18]. Multiple studies confirmed that elevated Lp(a) levels are a predictor for major adverse cardiovascular events following PCI (Table 2) [18].

In 2022, the European Atherosclerosis Society (EAS) published a statement confirming the association between high Lp(a) levels and ASCVD and aortic valve stenosis, even with low LDL levels [29]. After reviewing the data, they concluded that Lp(a) elevation is associated with MI, strokes, and peripheral arterial disease in a primary prevention setting. The EAS also consolidated many theories about the pathogenic mechanisms of Lp(a) elevation with regard to ASCVD, stating that Lp(a)’s pro-inflammatory mechanisms can be attributed to the following: Lp(a)’s cell signalling properties, its affinity for oxidised phospholipids, and its ability to promote pro-atherosclerotic chemical synthesis and secretion [29].

The AIM-HIGH sub-study reported Lp(a) to be associated with carotid plaques and mural thrombi in 214 trial participants, leading the authors to suggest that “Lp(a), HDL-C, or ApoA1 [are] independent factors associated with high-risk plaque features” [30]. Of note, Lp(a) levels do not appear to increase the risk of venous thrombosis or affect fibrinolysis, with data from the Copenhagen City Heart Study and the Copenhagen General Population Study showing no correlation between Lp(a) levels and the risk of venous thrombosis [31].

## 6. Measurement of Plasma Lp(a) Concentration and Standardisation

Circulating plasma Lp(a) levels rise after birth, reaching a constant concentration in the first months of life [1,32]. Thereafter, individual Lp(a) concentrations remain relatively stable throughout life, ranging widely from <1 to 200 mg/dL in the general population [33]. Many studies show women to be more prone to increased Lp(a) concentrations [34,35], and there are racial differences also reported, with Lp(a) being lowest in Caucasian patients and highest in patients of African ethnicity [36]. The emergence of Lp(a) as a risk factor for cardiovascular disease has led to recommendations for measuring it at least once during lifetime, especially in high-risk populations [37,38]. However, there is controversy regarding the standardisation and validity of methods used to measure Lp(a) concentrations due to the highly variable size of Lp(a), which is largely determined by the apo(a) size. The resulting inter-individual and intra-individual variability in different populations is a consequence of the fact that most individuals are carriers of two different apo(a) alleles [39,40]. This variability has resulted in different methodologies for Lp(a) measurement—either measurement of molarity of Lp(a) or quantification of the mass concentration of Lp(a). The latter is more prone to variation, leading to diverging values of different mass-targeting kits, even within the same population with a standard Lp(a) molar concentration [41,42]. Despite this issue and the apo(a)-insensitive quantifying methods [42,43], commercial kits measuring Lp(a) in mg/dL instead of estimating its molarity in nmol/L are still frequently referenced in practice and the literature [44,45]. It is important to note that converting the mass concentration of Lp(a) to its molar equivalent (mg/dL to nmol/L) cannot produce accurate results, despite attempts suggesting an approximate 2–2.5× conversion factor [46].

## 7. Use of Pharmaceutical Agents to Reduce Lp(a) Levels

Lp(a) concentration is primarily under genetic control, with the modest influence of environmental factors. Pharmacologic and apheresis strategies to reduce Lp(a) levels have potential therapeutic importance. Lipid-modifying therapies, including statins, ezetimibe, and fibrates, reduce cardiovascular risk without substantially affecting Lp(a) levels. Conversely, lipid-modifying therapies such as niacin and CETP inhibitors lower Lp(a) levels without substantially influencing cardiovascular risk. PCSK9 monoclonal antibodies are the first class of pharmacologic agents to lower Lp(a) levels and substantially reduce cardiovascular risk. The clinical benefit of PCSK9 monoclonal antibodies is associated with baseline and on-treatment levels of Lp(a), but this effect is likely due to the dramatic reductions in LDL cholesterol.

Conflicting data exist regarding the effect of statins on Lp(a). Several studies show that the current “gold-standard” treatment to lower LDL cholesterol levels (statins) increases Lp(a) levels [32,44]. Although statins remain one of the most effective and safest drug categories for atherosclerotic cardiovascular disease prevention, one study reported a mean 11% increase in Lp(a) levels with their use [47,48,49]. The ILLUMINATE trial revealed that, in high-risk CVD patients, Lp(a) levels were positively and dose-dependently correlated with atorvastatin dose [50]. These data should be tempered by data from meta-analyses on the impact of different types and dose regimens of statins, showing no clinically significant reduction in Lp(a) levels [51]. Despite this at best neutral effect of statins, the European Atherosclerosis Society consensus statement suggests that statin therapy should not be discontinued, as their cardioprotective actions overcome any risks associated with increased Lp(a) plasma concentrations [34].

As with statins, the data on the effect of ezetimibe on Lp(a) circulating levels are also inconclusive. A meta-analysis of seven randomised controlled trials reported ezetimibe to reduce Lp(a) by 7%, an effect considered ineffective in reducing the Lp(a)-related risk of CVD events [52]. Conflicting evidence was reported from a large meta-analysis from 10 randomised placebo-controlled clinical trials, demonstrating no effect of ezetimibe therapy on modulating plasma Lp(a) concentrations, either as a monotherapy or in combination with a statin [53].

Niacin (nicotinic acid) is an effective therapeutic agent for raising HDL levels and has been used to reduce CVD events and mortality [54,55]. Niacin acts by silencing apo(a) gene expression in hepatocytes and is an approved treatment for Lp(a) reduction. The effect of niacin is dose-dependent and leads to a 25% to 38% reduction in Lp(a) levels when niacin is administered at a 2 to 4 g daily dosage, respectively. Disappointingly, this reduction in Lp(a) levels has not been associated with any effect on CVD reduction [56]. Although a large meta-analysis of 14 randomised placebo-controlled clinical trials reported a significant reduction by 23% in plasma Lp(a) concentration, the prognostic relevance of this effect has yet to be clarified [57], while the Lp(a)-lowering effect of niacin has not been linked to any clinical benefit, in terms of ASCVD events, so far [58].

The failure of “first line” lipid-lowering therapies to show a reduction in Lp(a) levels, or in the case of niacin, the reduction in Lp(a) not being linked to CV event reduction, has led to the development and study of agents that target reductions in serum Lp(a) concentrations.

### 7.1. Apheresis

Currently, the most effective treatment for high Lp(a) levels is apheresis—filtering patients’ blood to selectively remove lipoproteins containing ApoB100. This treatment is well tolerated by patients and reduces both serum LDL and Lp(a) concentrations by 60–70%, thus reducing cardiovascular disease risk by 54–90% [59]. HEART UK guidelines recommend apheresis in patients with Lp(a) levels higher than 60 mg/dL, LDL-C levels above 125 mg/dL on maximally tolerated cholesterol-lowering therapy, and progressive coronary artery disease [60].

### 7.2. PCSK9 Inhibitors

PCSK9 inhibitors reduce serum LDL levels, but recent research shows that they are also effective in reducing serum Lp(a) concentrations [61]. With PCSK9 receptors inhibited, LDL is more readily absorbed into hepatocytes, thus decreasing serum LDL concentrations. In clinical trials, PCSK9 inhibitors reduce serum Lp(a) concentrations by up to 30% [61]. However, as these trials were not designed to observe changes in Lp(a) concentrations, we cannot assume that this Lp(a)-lowering effect is durable or clinically significant.

### 7.3. Fibrates

Fibrates also lower both serum cholesterol and Lp(a). Fibrates are synthetic ligands of peroxisome proliferator-activated receptor alpha (PPAR alpha). PPAR alpha is a transcription factor predominantly found in fatty-acid-metabolising tissues, e.g., liver and kidney tissue. PPAR alpha and fibrates enhance lipolysis in liver tissue by increasing lipoprotein lipase synthesis, whilst simultaneously inhibiting apoC-III gene expression. ApoB production is also decreased through fibrate action [62]. It has been shown in animal studies that free fatty acid uptake and catabolism is enhanced by fibrates, leading clinicians to the conclusion that fibrates promote the catabolism of cholesterol and Lp(a) in the liver, thus decreasing cardiovascular disease risk [62].

## 8. Trial Data of Targeted Lp(a) Reducing Agents

### 8.1. Pelacarsen

Pelacarsen is a promising antisense oligonucleotide therapy that reduces serum Lp(a) concentrations. This drug is an RNase H-competent antisense oligonucleotide (ASO). ASOs bind to complementary mRNA strands using the gapmer approach: RNA-based sequences “frame” a DNA-based gap, allowing mRNA to bind. RNase H1 then cleaves the RNA at the site of RNA binding [63]. The effect of Pelacarsen is enhanced almost 30-fold by adding GalNAc [64]. Pelacarsen is currently in phase 3 clinical trials expected to finish in May 2025. The results of early phase trials are promising: serum Lp(a) concentrations decreased by between 35% and 80% (*p* value range 0.003 to <0.001) with multiple administrations, with results lasting for up to 16 weeks [64]. Others, who received weekly doses of 20 mg of Pelacarsen, exhibited a reduction in Lp(a) levels of at least 50 mg/dL, with results persisting for at least 180 days post-administration [64].

### 8.2. siRNA

Short interfering RNA (siRNA) therapies, such as olpasiran and SLN360, are also in development [65]. SiRNA is double-stranded RNA, composed of antisense and sense strands. The antisense strand is complementary to the target mRNA sequence. Once the siRNA enters the cell, the antisense strand is incorporated into the RNA-induced silencing complex (RISC). The activated antisense strand binds to the target mRNA (in this case, LPA mRNA), signalling where argonaute proteins (e.g., AGO2) should cleave the target mRNA [66]. The target mRNA is then degraded and unable to synthesise proteins [37]. The antisense strand remains unchanged and can continue to degrade remaining copies of the target mRNA. Olpasiran activity is enhanced when conjugated by GalNAc. Olpasiran’s duration of action is prolonged, lasting on average between 3 and 6 months [41]. This is because the drug is renally excreted and is active in hepatocytes [67].

Olpasiran is currently undergoing clinical trials. Phase II trials, reinforced from animal studies, show a decrease in Lp(a) concentrations of over 90% at doses greater than 75 mg, with effects lasting for several months [68]. Phase III clinical trials investigating the effectiveness of olpasiran in reducing risk of cardiovascular events in patients with pre-existing atherosclerotic cardiovascular disease and elevated Lp(a) are currently underway and expected to be complete by December 2026 [63].

SLN360 is a GalNAc-modified siRNA that targets Lp(a) mRNA [69]. Through various clinical trials, it has been shown that it also decreases both total cholesterol and Lp(a) concentrations in humans inW a dose-dependent manner [70]. In a phase I trial, in adult patients with high Lp(a) and no known prior cardiovascular diseases, SLN360 reduced Lp(a) concentrations by more than 80% with doses of 600 mg, with effects persisting for at least 150 days. It was well tolerated, with the adverse effects reported being low-grade injection site events and headache. One patient also had elevated ALT and AST levels during the trial, which were attributed to the patient having the SARS-CoV-2 vaccine. SLN360 has been approved for phase II studies in patients with pre-existing ASCVD, with estimated completion in 2024 [63].

## 9. Future Implications

Although existing pharmacology appears promising in reducing Lp(a), we await data to show whether this can affect cardiovascular events. If proven, then the possibility of a permanent reduction in Lp(a) through gene therapy would become clinically appealing.

### CRISPR/Cas9

CRISPR/Cas9 involves a strand of antisense RNA and Cas9, an endonuclease. The antisense RNA binds to a section of complementary target DNA, which activates Cas9. Cas9 then makes double-stranded breaks in the DNA, creating blunt ends in the strand. The target DNA is then excised and “melted,” with the remaining DNA strands repairing themselves by binding the two blunt ends together [71]. As current research points to PCSK9 inhibiting both cholesterol and Lp(a) clearance, research is being conducted in mice and non-human primates to determine whether CRISPR/Cas9 genome editing is effective in permanently lowering serum Lp(a) levels [72]. Current research into CRISPR/Cas9 efficacy is promising. Musunuru et al. used nanoparticles with base editors and guide RNA in animals, showing a decrease in plasma PCSK9 levels of approximately 90%. Unlike previous studies, the side effects caused by the treatment—namely liver toxicity—were transient, and the duration and efficacy of the treatment outweighed these issues [73].

## 10. Conclusions

Lp(a) is considered a major risk factor for atherosclerotic CVD, with several studies confirming an association between elevated Lp(a) levels and incident CAD in the general population. Additionally, observational data suggest that Lp(a) is associated with increased adverse events in patients with established CAD and with aortic stenosis. Imaging data suggest that the mechanism of Lp(a) and worse CV outcomes may be due to the adverse effect of high Lp(a) levels on plaque vulnerability. Promising pharmacotherapy interventions show reductions in serum Lp(a) levels, but trial data of cardiovascular risk reduction are awaited.

## Figures and Tables

**Table 1:** Epidemiological studies suggesting a causal role of Lp(a) in CVD.

Epidemiological Studies	Patient Cohort	Study Design	Outcome	Results
Kamstrup, P.R., et al., 2007 [19]	9330 randomly drawn patients from general population cohort study	10-year follow up after blood sampling	Registry-based CV outcomes	Stepwise increase in risk of MI with increasing levels of lipoprotein(a), with no evidence of threshold effect
Kaltoft, M., et al., 2022 [20]	12,006 patients following CT 85,884 patients to examine risk of heart disease	Individuals who underwent cardiac computed tomography to measure mitral and aortic valve calcification and to examine risk of heart valve disease after blood sampling	Incidence of aortic and mitral valve disease	Elevated lipoprotein(a) was genetically and observationally associated with mitral and aortic valve calcification and aortic valve stenosis
Danesh, J., et al., 2000 [17]	5436—non-selected population	Meta-analysis of 27 prospective studies	Mean follow up of 10 years	Confirmed association between Lp(a) and CHD
Erqou, S., et al., 2010 [21]	11,396 patients with vascular disease and 46,938 controls	Meta-analysis of 40 prospective studies	Assess association of lipoprotein(a) isoforms with cardiovascular disease risk	People with smaller apo(a) isoforms have an approximately 2-fold higher risk of CHD or ischemic stroke than those with larger proteins
Pare, G., et al., 2019 [22]	6086 cases of first MI and 6857 controls from the INTERHEART study stratified by ethnicity and adjusted for age and sex	A total of 775 Africans, 4443 Chinese, 1352 Arabs, 1856 Europeans, 1469 Latin Americans, 1829 South Asians, and 1221 Southeast Asians were included	Incidence of MI	Lp(a) and isoform size varied markedly between ethnic groups. Higher Lp(a) associated with increased MI risk, with especially high population burden in South Asians and Latin Americans. Isoform size was inversely associated with Lp(a) but did not significantly contribute to risk

**Table 2:** Lp(a) as a predictor of MACE events following PCI.

Study	Study Population	Primary Endpoint	Results
Zhang, H., et al., 2023 [23]	Patients with PCI for ISR	MACE and repeat revascularisation	Increased risk of MACE and repeat revascularisation in high-Lp(a) group (HR: 1.31, CI: 1.08–1.58, *p* = 0.007)
Yoon, Y.H., et al., 2021 [24]	Patients with previous PCI	CV death, MI and ischaemic CVA, and recurrent ischaemic events	Increased ischaemic events in elevated-Lp(a) group (aHR: 1.17, CI: 1.05–1.30, *p* = 0.004)
Qin, S.Y., et al., 2013 [25]	Meta-analysis of patients with previous PCI, ISR, and de novo lesions	Rate of in-stent restenosis	Increased ISR in patients with elevated Lp(a) (SMD = 0.42, CI: 0.14–0.71, *p* = 0.003)
Liu, H., et al., 2022 [26]	Patients with low LDL following PCI	CV death; CVA, MI, and repeat revascularisation	Increased incidence of repeat revascularisation in high-Lp(a) group (13.3% vs. 6.9%, *p* < 0.05)
Park, S., et al., 2015 [27]	Stable angina patients post PCI	MI and revascularisation	Increased rate of restenosis (19.8% vs. 7.9%, *p* = 0.001)
Kimura, T., et al., 2022 [28]	Patient with PCI for de novo lesions	CV death, MI, stent thrombosis, and unplanned revascularisation	Higher incidence of MACE in Lp(a) > 30 mg/dL (33% vs. 15.9%, *p* < 0.001)

**Figure A1:**
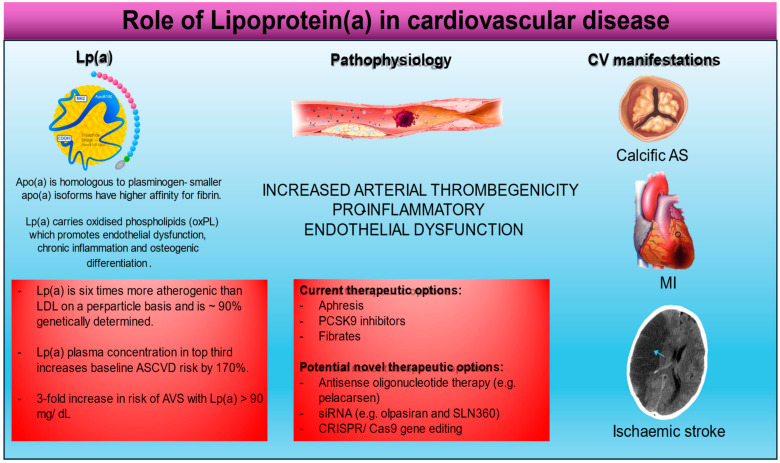
The role of lipoprotein(a) in cardiovascular disease.

## Abbreviations

CVD	cardiovascular disease
IHD	ischaemic heart disease
MI	myocardial infarction
LDL	low-density lipoprotein
oxPL	oxidised lipoprotein
ASCVD	atherosclerotic cardiovascular disease
PCI	percutaneous coronary intervention
MACE	major adverse cardiovascular events
CV	cardiovascular
AVS	aortic valve stenosis
CHD	coronary heart disease
ISR	in-stent restenosis
CVA	cerebrovascular accident
EAS	European Atherosclerosis Society
PCSK9i	proprotein convertase subtilisin/kexin type 9
PPAR alpha	peroxisome proliferator activated receptor alpha
ASO	antisense oligonucleotide
GalNAc	N-acetylgalactosamine
ALT	alanine transaminase
AST	aspartate transaminase
SARS-Cov-2	Severe acute respiratory syndrome coronavirus 2
CRISPR	clustered regularly interspaced short palindromic repeats
